# Resilience and hopelessness mediate the relationship between benevolent childhood experiences and life satisfaction: evidence from a cross-cultural study

**DOI:** 10.1186/s40359-024-02134-5

**Published:** 2024-11-07

**Authors:** Melih Sever, Oktay Tatlıcıoğlu, Telma Catarina Almeida, E. P. Abdul Azeez, Sónia Caridade, Olga Cunha

**Affiliations:** 1 Social Work Department, Suleyman Demirel University, Isparta, Turkey; 2 Postdoctoral Researcher at Sophia University, Tokyo, Japan; 3 Social Work Department, Dicle University, Diyarbakır, Turkey; 4 Egas Moniz Center for Interdisciplinary Research (CiiEM), Egas Moniz School of Health & Science, Caparica, Almada 2829-511 Portugal; 5 Department of Social Sciences, School of Social Sciences and Languages, Vellore Institute of Technology, Vellore, Tamil Nadu 632014 India; 6 Psychology Research Centre, University of Minho, Braga, Portugal

**Keywords:** Benevolent childhood experiences, Life satisfaction, Resilience, Hopelessness, Mediation model

## Abstract

**Background:**

A growing body of literature focuses on the role of benevolent childhood experiences (BCEs) in predicting adulthood well-being, in addition to adverse childhood experiences (ACEs). However, cross-cultural differences are generally ignored in this endeavor. Hence, this study aimed to explore the role of BCEs in predicting life satisfaction, resilience, and hopelessness. We also examined the potential of resilience and the role of hopelessness in mediating the relationship between BCE and life satisfaction.

**Methods:**

A total of 850 university students from Turkey (*n* = 371), Portugal (*n* = 248), and India (*n* = 231), aged 17 to 58 years (*M* = 22.12, *SD* = 4.41), participated in the study. Participants completed an online protocol consisting of measures to assess BCEs, life satisfaction, resilience, and hopelessness.

**Results:**

BCEs, hopelessness, and life satisfaction have significantly differed among the samples based on the country of residence. BCEs were positively correlated to resilience and life satisfaction and negatively to hopelessness. In the sequential mediation model, after controlling for country and sex, resilience and hopelessness sequentially mediated the relationship between BCEs and life satisfaction. BCEs were associated with life satisfaction, resilience, and hopelessness across countries and sexes. The model explains 42.8% of the variability.

**Conclusion:**

Despite differences between countries, BCEs are important predictors of adult well-being in all three countries and should be monitored along with ACE. Further, resilience seems to have an important role in lowering the negative consequences of lower BCEs and feelings of hopelessness, pointing to the need to strengthen psychological resilience among adults.

## Introduction

Researchers largely focused on pathological dimensions in studying individual well-being, neglecting the positive and protective factors till the recent past. With the development of positive psychology in the late 1990s centered on the study of the abilities and resources of individuals, in their positive characteristics, psychological assets, and strengths, a new paradigm of mental health analysis has been observed [1, 2]. Since then, we have witnessed a proliferation of positive psychology lenses to understand mental health and to enhance the resources of individuals to be resilient and flourish [3] rather than to reduce psychopathological symptoms [4]. This is contextual because it has been shown that specific positive individual characteristics (e.g., cognitive flexibility, optimism) or the environment (e.g., family support, adequate maternal care) seem to play a buffering role in the face of adversity, promoting people’s resilience, making them more capable of coping, controlling and dealing with stress and preventing the development of mental disorders [5].

In this context, the analysis of childhood experiences, both benevolent and adverse, seems to be equally important in addressing the mental health of individuals. In this sense, public health research has highlighted the important role of cumulative benevolent childhood experiences (BCEs) in their ability to modulate potential negative effects of adverse childhood experiences (ACEs) [6]. However, studies neglect the role of context and different childrearing and cultural practices in terms of psychosocial structures in different countries. Hence, in this study, we aimed to examine BCEs and other factors predicting well-being in a multinational sample to highlight the differences and similarities between contexts and to test a model that potentially mediates the relationship between BCEs and life satisfaction in our sample.

For example, a study by Gunay-Oge et al. [7] found that BCEs are positively linked to factors that reduce the risk of personality psychopathology and healthy personality development in a Turkish sample. In this sense, the authors highlighted the importance of improving and promoting BCEs and investing in preventing ACEs. A recent study by Almeida et al. [8] also reinforces the positive role of BCEs on the relationship between child maltreatment and affective lability. The ecological-transactional model seeks to explain the importance of early experiences for life span and adaptation or maladaptation, recognizing the role of BCEs as an important protective factor for victimization experiences [9, 10]. BCEs allow individuals to develop skills to respond positively to negative events, enhancing their ability to deal with adverse experiences [11] since individuals who experience BCEs are more inclined to develop better self-esteem [12] and emotional regulation skills [13].

### Benevolent childhood experiences

The BCEs include relational and internal safety and security, positive and predictable quality of life, interpersonal support and other community resources [14], healthy attachment bonds, and effective parenting behaviors [15]. Empirical evidence suggests that BCEs may play a significant role in adulthood and are associated with positive functioning in both a Portuguese sample [8, 16, 17] and a multicultural sample in the US [14]. For example, research shows the importance of early positive experiences in preventing mental health problems [8, 18]. Some studies concluded that BCEs are associated with less emotional lability [17], more health, social and relational skills [19], emotional regulation [20], and empathy in adulthood [21]. BCEs are negatively related to the risk of developing symptomatology, such as depression and anxiety. There is also an inverse dose-response relationship between the risk of depression and cumulative BCE exposure [18].

Positive interpersonal experiences with family and friends may lower the adverse effects of ACEs [19]. Even though both may exist, the BCEs can buffer negative experiences [8, 14, 16]. The BCEs are promotive factors that can reduce the risk of stress and psychopathology [14]. Therefore, these positive factors interact with the adverse ones, and the harmful effects of ACEs are lessened. The BCEs are essential in understanding developmental protective factors that guard against damaging effects [19]. Research has identified the moderating effect of BCEs on the impact of traumatic experiences, resulting in more ability to develop resilience when facing adverse experiences [8, 14, 22] and increasing and promoting well-being [18].

### Resilience, hopelessness, and life satisfaction

The ability to recover from sickness, psychological hardships, adverse events, or stress is known as resilience [23]. According to Fletcher and Sarkar [24], resilience is linked to two concepts, adversity, and adaptability, and it is a strength-based approach to stress. Resilient people may be able to deal with difficult situations and even thrive in hardship [25]. Resilience draws attention in higher education in terms of increasing academic success and resilience against stress [26]. An inverse correlation was found between depression and resilience in US college students who had ACEs [27]. High school students with high psychological resilience tend to experience less stress and anxiety and thus have higher life satisfaction in Norway [28]. As opposed to that, Alnıaçık et al. [29] found that psychological resilience has a negative direct effect on hopelessness and a positive direct effect on hope in a Turkish sample. Karagöz et al. [30] also found a positive relationship between psychological resilience and life satisfaction in their study with health professionals in Turkey.

Hope is an essential element of a happy and satisfying life. However, hopelessness among adults is prevalent and is significantly associated with lower overall well-being [31]. It is an emotional state involving pessimistic outlooks of the future that potentially disturbs the ability to reach one’s goal [32]. The state of hopelessness is significantly determined by various psychosocial factors and available literature evidence that, to some extent, childhood experiences play a crucial role [33]. For example, ACEs are associated with various psychopathological conditions, including depression [34]. A recent US study reports that BCEs are negatively correlated to depressive symptoms [11], in which hopelessness is a significant component. Although studies on the relationship between BCEs and psychopathology are available, research focusing on the role of BCEs, specifically on hopelessness, is not yet available in the current literature.

Life satisfaction is a significant indicator of well-being [35] and is defined as the overall positive attitude toward one’s life [36]. Higher life satisfaction is instrumental and associated with better mental health outcomes [37–39]. A study among university students in Korea indicated that life satisfaction is a protective factor in depressive symptoms and crucial in preventing depression [40]. Another study among South African university students highlights that better life satisfaction is linked to lower levels of hopelessness, a crucial symptom of depression [41]. However, more research is required to establish how factors like psychological resilience mediate the relationship between hopelessness and life satisfaction and how BCEs predict this relationship in different countries.

### Present study

Considering the findings on the potential association between BCEs and individuals’ well-being, the present exploratory study examines BCEs, hopelessness, resilience, and life satisfaction and their relationship across the samples from Turkey, Portugal, and India. Specifically, the role of BCEs in predicting life satisfaction, hopelessness, and resilience was investigated, as well as the potential differences between the countries in such variables. In this cross-sectional model, we attempt to understand whether measures like hopelessness and resilience mediate the relationship between BCE and life satisfaction across samples with different sociocultural backgrounds and childrearing practices. The selection of these three countries was based on convenience, guided by the availability of researchers interested in cross-cultural comparisons across varying cultural contexts.

Culture has been defined as “the set of attitudes, values, beliefs, and behaviors shared by a group of people, communicated from one generation to the next” [42]. Literature has found that culture shapes our daily lives with differences in religion, daily practices, education, work, language and interaction, food, discipline, hobbies, rituals, beliefs, social activities, and so on [43]. Therefore, cultural background seems to play a vital role in molding several aspects of our lives, such as childhood experiences, resilience, hope, and life satisfaction, while also holding the potential to hinder them [43]. Although all these factors might also be influenced by other factors such as socioeconomic status, societal influence, and healthy relationships, cultural beliefs and parental socialization expectations, attitudes, and practices significantly shape one’s childhood experiences, life satisfaction, resilience, and hope [44, 45]. For example, multi-country studies reported that cultural levels of individualism are correlated to well-being [35, 46, 47], possibly because individualism socializes people to be happier [48]. However, cultural aspects other than the culture’s individualist or collectivist nature might influence individuals’ satisfaction with life and overall well-being. Considering the case of Portugal, Turkey, and India, collectivist cultures, the Ipsos Global Happiness Survey [49] revealed significant differences between countries concerning happiness and life satisfaction, with India ranking 4th and Turkey 28th out of 32 countries. Portugal occupies the 11th place in the ranking. These findings suggest that different cultural aspects may be linked to the individuals’ functioning as they may influence other important aspects. Research focused on place has shown that individuals from low-income communities have less access to educational and social capital than their wealthier counterparts [50]. Thus, their cultural background also shapes individuals’ positive (and negative) experiences. In addition, it is also known that culturally and contextually specific aspects contribute to individuals’ resilience and that the influence of resilience on individuals’ lives depends on the specific culture and context in which resilience is realized [51]. Research has also shown that individuals’ cultural context is related to hope and may operate differently in different cultural groups [52].

**Fig. 1:**
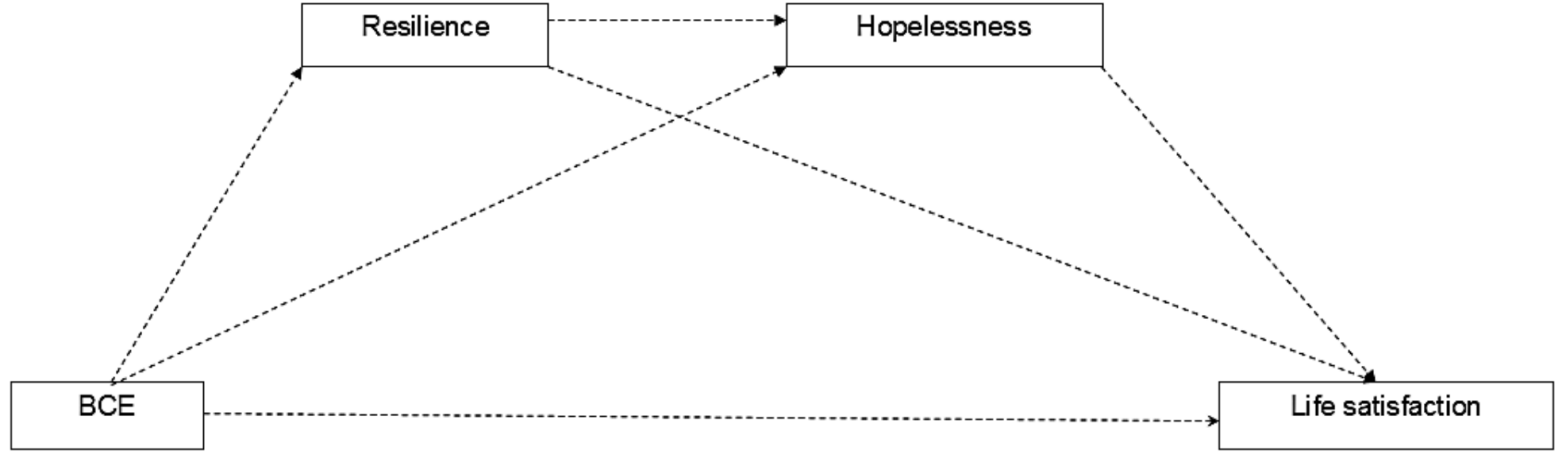
Mediation model of resilience and hopelessness in the relationship between benevolent childhood experiences and life satisfaction

Portugal, Turkey, and India are all collectivist cultures, but they have diverse and, to some degree, dissimilar psychosocial structures. Differences between the groups were also found in terms of public health care, education expenditures, Gross Domestic Product per capita, life expectancy at birth, school life expectancy, and employment rate. To our knowledge, no studies have analyzed the differences in terms of childhood experiences, life satisfaction, resilience, and hopelessness between countries. Based on the theoretical and empirical background mentioned above, this study proposes a conceptual model (Fig. 1) to link these variables with life satisfaction. It is hypothesized that BCEs exerted a direct influence on life satisfaction and an indirect influence mediated by resilience and hopelessness. This study aims to provide a new insight into the mediating role of resilience and hopelessness between BCEs and life satisfaction among university students from Portugal, India, and Turkey.

## Methods

### Sample

This cross-sectional and cross-cultural descriptive study analyzes survey data from three countries. Inclusion criteria included: (a) being a university student in Portugal, India, or Turkey and (b) having Portuguese, Indian, or Turkish nationality, respectively. University students from Portugal (*n* = 248), India (*n* = 231), and Turkey (*n* = 371) participated in this research and filled out an online survey in Portuguese, English, and Turkish, respectively. The sample was mainly composed of female students (*n* = 611, 71.9%), with an average age of 22.12 years old (*SD* = 4.41), ranging between 17 and 58 years.

### Procedures

University students from Turkey, India, and Portugal completed identical surveys in their respective languages (Turkish, English, and Portuguese) in March-September 2022. The original versions of the instruments were utilized with the Indian sample. At the same time, participants from Turkey and Portugal responded to adapted versions of the same scales in Turkish and Portuguese, respectively. The research team in the respective countries collected the data from the participants through online forms. The research link was shared with the university students via institutional email using the different universities’ mailing lists. In addition, the study was presented in the classes by the researchers, appealing to students’ participation. The participants accessed the link shared with them and answered the research protocol.

Participation in the study was voluntary and anonymous. All the participants signed an electronic informed consent form to complete the surveys. No course credit or compensation for their time was provided for participants in all countries.

### Measures

#### Demographic variables

A demographic questionnaire was used to collect the following information: age, gender, and country of origin.

#### Life satisfaction

Diener et al. [53] developed the Satisfaction with Life Scale (SWLS) to measure life satisfaction. It is a widely used scale for assessing one’s subjective life satisfaction. SWLS is a five-item single-factor scale with a response range of 1 to 7 (1 strongly disagree to 7 strongly agree) (e.g., “So far I have gotten the important things I want in life”). The scale has good psychometric properties [53]. The original version was used among the Indian sample in this study. Dağlı and Baysal [54] worked on the Turkish adaptation of the scale. They converted the scale into a 1–5 response range in the Turkish version, revealing good psychometric properties. The Portuguese version also has a 5-point item scale, revealing good internal consistency [55]. The internal consistency in the present study was good (*α* = 0.83).

#### Benevolent childhood experiences

The Benevolent Childhood Experiences scale (BCE) is a 10-item questionnaire that assesses pleasant childhood experiences from birth through age 18 [14]. Items cover various aspects of childhood development, including reassuring beliefs, trusting providers, and consistent family routines (e.g., “Did you have at least one caregiver with whom you felt safe?“). The original version of the scale was used among the Indian sample. The original version revealed good psychometric properties. Oge et al. [56] worked on the Turkish version, finding an internal consistency of 0.61. The Portuguese version [16] also reached good psychometric properties. In this sample, the internal consistency was 0.69.

#### Hopelessness

Beck [57] created the hopelessness scale (Beck’s Hopelessness Inventory), which assesses pessimism. The measure has 20 “true/false ‘’ dichotomous prompts divided into three categories: future thoughts, motivation, and expectations (e.g., “My future seems dark to me.“). The total scores were computed by reversing nine items and adding the item scores together. This study employed two sub-factors of the 12-item scale (expectations and ideas about the future). Pessimism is associated with higher total scores (range 12–24). The original English version of the scale is used among the Indian sample. Seber et al. [58] worked on the Turkish adaptation, finding a Cronbach alpha of 0.86. The Portuguese version was developed by Cruz [59] and revealed good psychometric properties. In the current study, the internal consistency was .70.

#### Resilience

Smith et al. [23] created the Brief Resilience Scale (BRS) to evaluate one’s capacity to deal with instabilities and challenges throughout life. BRS is a 5-point Likert scale (i.e., “Strongly disagree” (1), “Disagree” (2), “Neutral” (3), “Agree” (4), and “Strongly agree” (5) (e.g., “I usually come through difficult times with little trouble.“). High scores indicate a high level of psychological resilience. Almeida et al. [17] translated and adapted the Portuguese version. The Turkish version was adapted by Doğan [60], and the internal consistency was 0.83. The original version of the questionnaire was administered to the Indian sample. The current sample revealed an internal consistency of 0.73.

### Data analysis

Statistical data analyses were performed using IBM SPSS version 28.0 software. Descriptive analyses were conducted for sample characterization and main data description, with mean, standard deviations, ranges, and frequencies. Inferential statistical analyses were conducted through One-way ANOVA to explore the differences between participants from the three countries, and a *t*-test was conducted to analyze differences between sexes in the main variables. Effect sizes were computed using Eta Squared (η^2^) for one-way ANOVA and Cohen’s *d* for *t*-tests. Eta Squared was interpreted based on the following guidelines: 0.01 = small effect, 0.06 = medium effect, and 0.14 = large effect. The following guidelines were used to interpret Cohen’s *d*: 0.2 = small effect, 0.5 = medium effect, and 0.8 = large effect. Pearson’s correlation coefficients were also performed to assess the correlation between all variables in the study (i.e., benevolent childhood experiences, life satisfaction, resilience, and hopelessness) and test the statistical assumptions (correlation coefficients between all variables) for the mediational analysis. Then, the mediational model was tested with Model 6 from PROCESS macro 4.1 for IBM SPSS software [61], using Bootstrapping Confidence Intervals. According to Hayes [61], a mediational model is theoretically based on a variable (i.e., number of BCEs) that predicts an outcome (i.e., life satisfaction) through a mediator variable (i.e., resilience and hopelessness) after controlling for sex and country. Thus, it is possible to establish two pathways by which BCEs may predict life satisfaction (a direct and an indirect pathway) [61]. The indirect effects were calculated with 5000 bootstrap samples and 95% Bias-Corrected Bootstrap Confidence Intervals (95% BCBCI) [62]. An effect is regarded as significant if the CIs do not include zero.

### Ethical considerations

This study has strictly adhered to the ethical principles of conducting research with human subjects. Participants voluntarily agreed to participate and gave their informed consent via online form. Lusófona University Ethical and Deontological Board, Portugal, and Dicle University, Turkey (253463) granted necessary institutional ethical committee approvals to conduct the study.

## Results

The mean difference based on country of residence and sex for the main variables is presented in Table 1. There were statistically significant differences between countries in BCEs, *F*(2, 847) = 42.67, *p* < .001, life satisfaction, *F*(2, 847) = 78.36, *p* < .001, and hopelessness, *F*(2, 847) = 216.92, *p* < .001, with medium to large effect sizes. Scheffe post-hoc tests revealed differences in BCEs between Portuguese and Turkish participants (*p* < .001) and between Turkish and Indian participants (*p* < .001), with the Indian students having the lowest scores. Regarding life satisfaction, Scheffe post-hoc tests showed statistically significant differences between Portuguese and Turkish participants (*p* < .001) and between Turkish and Indian participants (*p* < .001). Turkish participants revealed the lowest scores on life satisfaction. Finally, concerning hopelessness, post-hoc tests revealed differences between Portuguese and Turkish students (*p* < .001), between Portuguese and Indian students (*p* < .001), and between Turkish and Indian students (*p* < .001). Turkish students showed the highest scores, and the Indians had the lowest scores.

**Table 1:** Group differences for the main variables

Variables	Portugal (*n* = 248)	Turkey (*n* = 371)	India (*n* = 231)	F	*p*	η^2^
	M (SD)	M (SD)	M (SD)			
BCEs	8.87 (1.57)	7.59 (2.04)	6.60 (1.70)	42.67	< 0.001	0.092
Resilience	3.01 (0.69)	2.95 (0.89)	3.02 (0.65)	0.706	0.494	0.002
Hopelessness	2.38 (2.19)	5.03 (2.03)	1.82 (1.90)	216.92	< 0.001	0.339
Life satisfaction	17.23 (3.86)	13.64 (4.06)	17.03 (4.19)	78.36	< 0.001	0.156
	**Women** (***n*** = 611)	**Men** (***n*** = 239)		***t***	***p***	***d***
	**M (SD)**	**M (SD)**				
BCEs	8.32 (1.88)	8.02 (1.99)		2.070	0.039	0.158
Resilience	2.91 (0.76)	3.19 (0.78)		-4.761	< 0.001	0.363
Hopelessness	3.33 (2.51)	3.53 (2.53)		-1.073	0.284	0.082
Life satisfaction	15.60 (4.32)	15.74 (4.59)		− 0.140	0.889	0.011

Note. BCEs = Benevolent Childhood Experiences

Statistically significant differences were also found between sexes in BCEs, *t*(848) = 2.070, *p* = .039, and resilience, *t*(848) = -4.761, *p* < .001. Women reported higher scores on BCEs but lower scores on resilience (see Table 1).

Pearson’s correlation coefficients for all variables in the study are presented in Table 2. It was possible to verify that BCEs were positively and significantly correlated to resilience and life satisfaction and negatively and significantly correlated with hopelessness. Resilience was positively and significantly correlated to life satisfaction and negatively and significantly to hopelessness. Hopelessness was negatively and significantly correlated to life satisfaction.

**Table 2:** Pearson’s correlation coefficients for the main variables

	1	2	3
1. BCEs	1		
2. Resilience	0.221***	1	
3. Hopelessness	− 0.440***	− 0.273***	1
4. Life satisfaction	0.394***	0.355***	− 0.610***

Note. *** *p* < .001; BCEs = Benevolent Childhood Experiences

**Fig. 2:**
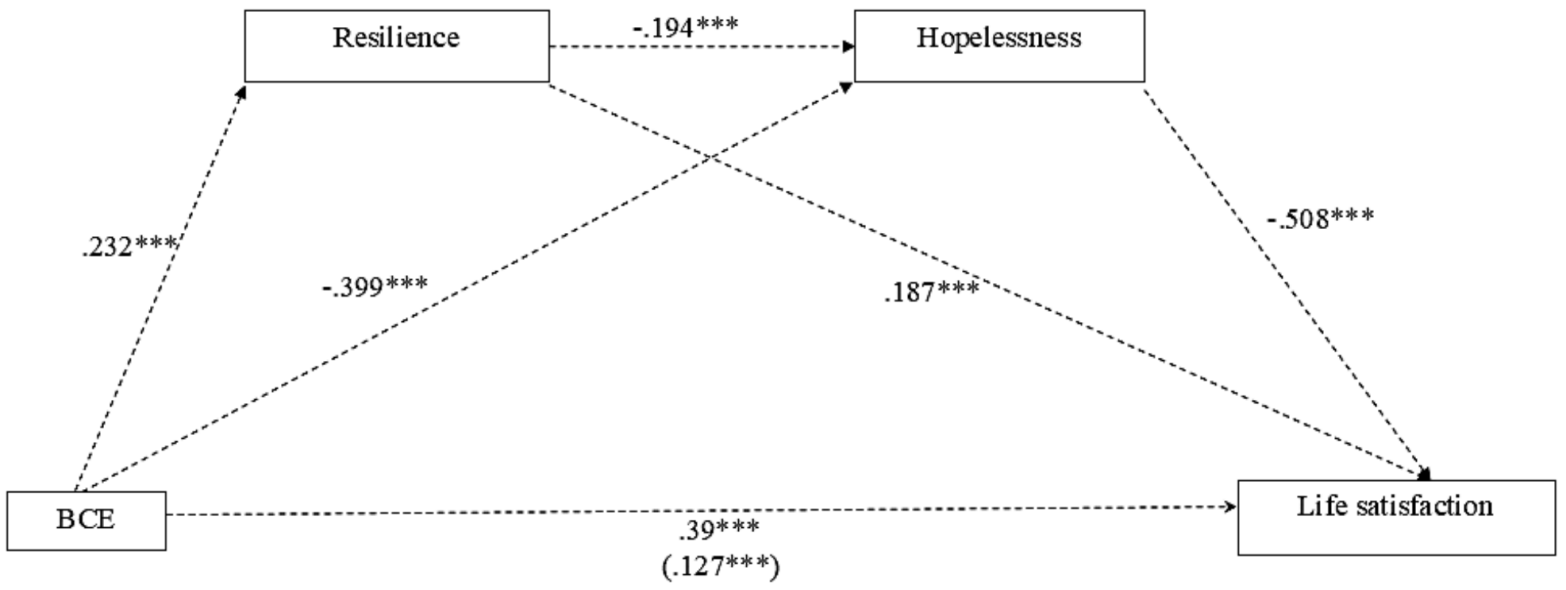
Mediation model of resilience and hopelessness in the relationship between benevolent childhood experiences and life satisfaction, controlling for country of origin and sex (*n* = 850) *** *p* < .001

Model 6 of the PROCESS macro was used to test the mediation effect of the studied variables. The mediation model explained 42.8% of the variance of life satisfaction, which was significant, R2 = 0.428, *F*(4,845) = 157.996, *p* < .001, after controlling for country and sex (see Table 3). BCEs significantly and positively predict life satisfaction, *b* = 0.909, *SE* = 0.073, *t* = 12.496, *p* < .001, 95% BCBCI 0.766, 1.052. BCEs have a significant and positive association with resilience, *b* = 0.094, *SE* = 0.013, *t* = 7.022, *p* < .001, 95% BCBCI 0.068, 0.121, which, in turn, predicts positively life satisfaction, *b* = 1.056, *SE* = 0.157, *t* = 6.719, *p* < .001, 95% BCBCI 0.748, 1.365. BCEs have a significant but negative correlation with hopelessness, *b* = -0.524, *SE* = 0.041, *t* = -12.854, *p* < .001, 95% BCBCI − 0.603, -0.444, which, in turn, negatively correlates with life satisfaction, *b* = − 0.888, *SE* = 0.052, *t* = -17.042, *p* < .001, 95% BCBCI − 0.991, − 0.786 (see Fig. 2). Life satisfaction positively correlates with resilience, *b* = 1.056, *SE* = 0.157, *t* = 6.719, *p* < .001, 95% BCBCI 0.748, 1.365.

**Table 3:** Regression coefficients, standard errors, and model summary information

	Resilience			Hopelessness			Life satisfaction		
	Coeff.	SE	*p*	Coeff.	SE	*p*	Coeff.	SE	*p*
BCEs	0.094	0.013	< 0.001	− 0.524	0.041	< 0.001	0.292	0.068	< 0.001
Resilience				− 0.629	0.102	< 0.001	1.056	0.157	< 0.001
Hopelessness							− 0.888	0.052	< 0.001
Country	− 0.023	0.035	0.631	− 0.311	0.103	0.001	− 0.336	0.157	0.033
Sex	0.315	0.058	< 0.001	0.348	0.175	0.047	0.143	0.266	0.591
Constant	2.148	0.122	< 0.001	9.827	0.418	< 0.001	13.342	0.815	< 0.001
	R^2^ = 0.081 *F*(3,846) = 24.734, *p* < .001			R^2^ = 0.238 *F*(4,845) = 65.910, *p* < .001			R^2^ = 0.428 *F*(5,844) = 126.348, *p* < .001		

Note. BCEs = Benevolent Childhood Experiences

Furthermore, results indicate some significant indirect effects. BCEs significantly indirectly predicted life satisfaction through resilience, *b* = 0.100, *SE* = 0.021, 95% BCBCI 0.062, 0.142, meaning that resilience partially mediates the relationship between BCEs and life satisfaction. BCEs also had a significant indirect effect on life satisfaction through hopelessness, *b* = 0.465, *SE* = 0.046, 95% BCBCI 0.378, 0.558. These results confirm that hopelessness partially mediated the relationship between BCEs and life satisfaction. Finally, an indirect effect of BCEs on life satisfaction through resilience and hopelessness, *b* = 0.053, *SE* = 0.012, 95% BCBCI 0.031, 0.079, was also found. Thus, resilience and hopelessness partially mediated the relationship between BCEs and life satisfaction.

## Discussion

This study explored the potential relationship between BCEs, life satisfaction, psychological resilience, and hopelessness in a multicultural sample. We attempted to develop a mediation model that explains how resilience and hopelessness mediate the relationship between BCEs and life satisfaction. Considering that the analysis of childhood experiences, positive and/or adverse, is fundamental in approaching individuals’ mental health, the present study is pertinent and relevant in understanding the role of BCEs in promoting individual well-being in different contexts [6].

Although the total sample showed a precise model, the study results revealed statistically significant differences in BCEs, life satisfaction, and hopelessness between the samples of the countries, with medium to large effect sizes, confirming our first hypothesis. Specifically, differences in BCEs between the Turkish and Portuguese participants and between Turkish and Indian participants were found, revealing lower BCE levels among Indian participants. The lower BCEs among the Indian sample point to the higher prevalence of ACEs, neglect, and abuse reported in the literature [63–65]. The lower GDP per capita, the higher population density, the low life expectancy at birth, and the lower school life expectancy observed in India compared to Portugal and Turkey are also some factors that could explain the differences found. However, no attempts have been made in the literature to compare these variables between Portugal, Turkey, and India. Additionally, a recent study exploring BCEs among multinational samples did not document country-specific differences [66]. Results also revealed statistically significant differences between countries concerning life satisfaction. In this measure, the Turkish sample showed the lowest score. Previous studies conducted among Turkish university students were also documented concerning life satisfaction levels [67]. Besides, the Ipsos Global Happiness Survey [49] posits Turkey in 28th out of 32 countries, while India and Portugal revealed more satisfaction with life. The trend remained the same in hopelessness as statistically significant differences were found between countries, with the Turkish sample showing the highest scores on hopelessness. While Kiral Uçar et al. [68] did not find significant differences in hopelessness levels between samples from six countries in their study, we argue that the ongoing economic turmoil, rising unemployment rates, and devaluing currency Turkey has been witnessing over the last few years may explain the results [69]. Indeed, Turkey’s unemployment rate is the highest of the three countries, and its GDP per capita is low (although slightly higher than that of India). However, unlike other variables, psychological resilience has not demonstrated a significant difference based on the participant’s country of origin. This result suggests that, in this specific study, the cultural background does not predict individuals’ resilience. However, the sample characteristics (i.e., university students) might also explain the absence of differences as students’ heterogeneous samples are not representative of the general public [70].

Statistically significant differences were also found between sexes in BCEs, with women reporting higher scores on BCEs than men despite the small effect size. Redican et al. [71] also found that females reported higher levels of BCEs in their large-scale representative study. A possible explanation for these gender differences in BCEs could be related to social gender norms and expectations. Indeed, research revealed that women are often socialized to be caretakers, nurturers, and empaths from an early age, which can promote more positive childhood experiences related to benevolence [72]. These gender differences in BCEs could also be attributed to how men and women perceive interpersonal relationships, especially in the Turkish and Indian samples, where gender roles are mainly determined by patriarchal norms [73, 74]. In this sense, it has been argued that women value emotional connection with other people [75], and men mainly favor autonomy and individuality [76]. Differences in personality between men and women may provide another framework for understanding gender differences at the BCE level, as women tend to present higher levels of neuroticism, agreeableness, and extroversion [77]. Results revealed that women reported lower scores on resilience than men, following a previous meta-analysis [78]. Differences in socialization between men and women can also help to understand the lower scores on women’s resilience if we consider the evidence that men are generally more action-oriented, assertive, and focused on problem-solving [79]. In contrast, women tend to ruminate when distressed and may dismiss a lesser sense of mastery in their lives [80]. Nonetheless, these findings should be analyzed carefully since the magnitude of the effect is small.

The study shows that BCEs are significantly related to hopelessness. Participants who reported low BCEs had potentially high levels of hopelessness. This underlines how low BCEs are negatively correlated to hopelessness. People who did not have BCEs are likely to have hopelessness and other depressive features in later life. The available evidence that explored BCEs and depressive symptoms among different samples is also consistent with the present study’s results in supporting that greater BCE contributes to lesser depressive symptoms, including hopelessness [11]. However, the present study’s results also underline that psychological resilience is a protective factor in life satisfaction by mediating the relationship between BCEs and hopelessness. This finding uniquely contributes to the knowledge base on one important mediating factor between BCEs and hopelessness, as no other study has found this to our knowledge. However, our results should be carefully interpreted as we used a cross-sectional approach, and mediation analyses of longitudinal data among cross-sectional designs often generate biased estimates [81].

We found that in our total sample, participants with high self-reported BCEs also have high levels of psychological resilience. This result is consistent with studies underlining the BCEs’ role in positively predicting resilience-building features [22]. This result is consistent with a study conducted in Turkey [56], and no other studies have established this relationship to our knowledge. Participants who self-reported lower psychological resilience had higher levels of hopelessness. Studies among different populations also have shown that psychological resilience is negatively related to hopelessness [82–84].

Higher hopelessness is hypothesized to be associated with low life satisfaction among our multinational sample. The relationship between hopelessness and life satisfaction is negative. Participants who reported higher levels of hopelessness were also poor at life satisfaction. Consistent with this result, adolescent and student studies in Turkish and Hong Kong samples report that life satisfaction is associated with a lower risk of developing hopelessness [85, 86]. Another study involving university students underlined that lower levels of hopelessness are correlated to greater life satisfaction [87]. Moreover, psychological resilience mediates the relationship between hopelessness and life satisfaction. The finding on the mediating role of psychological resilience in depressive symptoms [88] and life satisfaction [89] is also documented in Chinese and Turkish samples in the previous literature.

BCEs are positively associated with resilience, and resilience relates to lower hopelessness, which then links to higher life satisfaction in young adults in our sample. This model suggests that psychological resilience acts as a protective factor in lessening the adverse consequences of hopelessness. Also, act as a promotive factor in the presence of BCEc on measures like life satisfaction. Kelifa et al. [90] tested a similar model in which resilience and depression mediated the relationship between ACEs and well-being in Eritrean college students, and the findings supported their model. Similar trends of mediating and moderating roles of resilience in buffering the negative psychological consequences have been mentioned in the literature [91–93]. However, our study is the first to report the potential of resilience in the relationship between BCEs and life satisfaction. In addition, and as previously mentioned, these results should be interpreted with caution as mediation analyses among cross-sectional designs should provide biased data [81].

## Limitations and future research

This research has some limitations that should be addressed. Firstly, data collected from three countries can only partially represent their regions and universities. Secondly, data collection was conducted through online tools, with the participation of volunteer students who accessed the survey link and completed all questions, as they were mandatory. Our study may have been affected by selection bias due to some factors. First, we did not track participants who discontinued the survey midway. As a result, we lack information about their characteristics (e.g., sociodemographic details) and the number of such participants, which may have influenced the results. Second, students who chose not to participate in the survey are also unknown to us in terms of their number and characteristics. In addition, we could not calculate the exact number of emails sent to potential participants, so the actual response rate is unknown.

Further, the findings of this study are limited by the use of a convenient sample composed solely of university students. As a result, the findings cannot be generalized beyond the specific participants in this study. The characteristics of the students who participated may differ from those who opted not to, further restricting the applicability of these findings to a broader context, including other university students, settings, or the general population. Besides, as one of the important limitations of this research, we should note the potential for bias in results inherent to cross-sectional mediation analyses [81], as we cannot infer causal relationships. Longitudinal data may be necessary to establish a more robust mediation model and draw rigorous conclusions. We suggest that future studies focus on positive aspects of childhood experiences through qualitative diaries, observation, and cohort studies.

## Conclusion

This is the first study to test the association between BCEs and life satisfaction through resilience and hopelessness across three countries. Strikingly, the Turkish scores significantly differed from the Portuguese and Indian samples. The analyses revealed that Turkey scored lower on life satisfaction and higher on hopelessness. Researchers and practitioners should address this issue holistically to understand why young adults from Turkey have low life satisfaction and high levels of hopelessness. India scored the poorest in BCEs, and researchers should investigate the nonpositive childhood experiences in India further.

This study found that BCEs are positively related to life satisfaction in young adults and should be monitored along with ACEs. Resilience might act as a protective factor against hopelessness, and hope also contributes to life satisfaction. The results revealed that hopelessness and resilience mediate the relationship between BCEs and life satisfaction, regardless of country-specific context. Thus, further investigation in different countries may reveal a global pattern.

## Data Availability

The data will be made available upon request by contacting the corresponding author.

## Abbreviations

BCE: Benevolent Childhood Experiences
ACE: Adverse Childhood Experiences
